# Frailty trajectories in community‐dwelling older adults during COVID‐19 pandemic: The PRESTIGE study

**DOI:** 10.1111/eci.13838

**Published:** 2022-07-28

**Authors:** Alberto Pilotto, Carlo Custodero, Sabrina Zora, Stefano Poli, Barbara Senesi, Camilla Prete, Erica Tavella, Nicola Veronese, Elena Zini, Claudio Torrigiani, Carlo Sabbà, Alberto Cella, the PRESTIGE Study Investigators, Marina Barbagelata, Lisa Annunziata Cammalleri, Romina Custureri, Simone Dini, Marcella Fama, Paola Giannoni, Valeria Pandolfini, Annamaria Piana, Alessandra Pinna, Martina Vigo, Erica Volta

**Affiliations:** ^1^ Geriatrics Unit, Department of Geriatric Care Orthogeriatrics and Rehabilitation Genoa Italy; ^2^ Department of Interdisciplinary Medicine, Clinica Medica e Geriatria “Cesare Frugoni” University of Bari “Aldo Moro” Bari Italy; ^3^ Department of Education University of Genoa Genoa Italy; ^4^ Department of Geriatrics University of Palermo Palermo Italy

**Keywords:** community, comprehensive geriatric assessment, COVID‐19, frailty, multidimensional prognostic index

## Abstract

**Background:**

Frailty has been recognized as potential surrogate of biological age and relevant risk factor for COVID‐19 severity. Thus, it is important to explore the frailty trajectories during COVID‐19 pandemic and understand how COVID‐19 directly and indirectly impacts on frailty condition.

**Methods:**

We enrolled 217 community‐dwelling older adults with available information on frailty condition as assessed by multidimensional frailty model both at baseline and at one‐year follow‐up using Multidimensional Prognostic Index (MPI) tools. Pre‐frail/frail subjects were identified at baseline as those with MPI score >0.33 (MPI grades 2–3). Frailty worsening was defined by MPI difference between 12 months follow‐up and baseline ≥0.1. Multivariable logistic regression was modelled to identify predictors of worsening of frailty condition.

**Results:**

Frailer subjects at baseline (MPI grades 2–3 = 48.4%) were older, more frequently female and had higher rates of hospitalization and Sars‐CoV‐2 infection compared to robust ones (MPI grade 1). Having MPI grades 2–3 at baseline was associated with higher risk of further worsening of frailty condition (adjusted odd ratio (aOR): 13.60, 95% confidence interval (CI): 4.01–46.09), independently by age, gender and Sars‐CoV‐2 infection. Specifically, frail subjects without COVID‐19 (aOR: 14.84, 95% CI: 4.26–51.74) as well as those with COVID‐19 (aOR: 12.77, 95% CI: 2.66–61.40, *p* = 0.001) had significantly higher risk of worsening of frailty condition.

**Conclusions:**

Effects of COVID‐19 pandemic among community‐dwelling frailer individuals are far beyond the mere infection and disease, determining a significant deterioration of frailty status both in infected and non‐infected subjects.

## 1 BACKGROUND

Frailty is a potentially reversible geriatric condition characterized by a reduction of biological reserves that predispose to countless negative outcomes including disability and mortality.^1^ With the extended life expectancy and the rapid increase of aging population, assessment of frailty status may represent an useful proxy to measure biological age, beyond simple chronological age.^2^ The divergencies between these two perspectives on patient's age have become particularly evident during the COVID‐19 pandemic.^3^, ^4^ Despite the initial epidemiological data suggested COVID‐19 as a geriatric condition with worst prognosis in older and multimorbid subjects,^5^ such ageistic criterion was progressively reviewed and overcome.^4^ Patients with same age could have completely different predisposition to contract Sars‐CoV‐2 and to experience severe consequences of the disease. Rather, it emerged that frailty is a better predictor of disease severity and higher incidence of negative outcomes in hospitalized older patients (e.g. mortality at short‐ as well as long term, length of hospital stay, higher incidence of admission to intensive care units and need of invasive mechanical ventilation).^6^, ^7^, ^8^, ^9^, ^10^, ^11^, ^12^, ^13^ Frail subjects may commonly experience atypical presentation of the COVID‐19 disease including hypotension, sudden functional decline, falls, and delirium, which may lead to diagnostic delay and further spread of infection.^14^

Also indirect effects of COVID‐19 pandemic, mainly related on restriction measures, have been extensively studied including the obvious difficulties in the access to care for subjects with chronic diseases, as well as with the impressive increased incidence of psychosocial disorders (e.g. depression, anxiety and loneliness), malnutrition (over‐ or under‐nutrition) and cognitive impairment, which may contribute to incidence and progression of frailty condition.^15^, ^16^, ^17^ However, there is still a paucity of direct evidence on the impact of COVID‐19 outbreak on frailty status in a population‐based setting. Recently a novel conceptual model for frailty evaluation has been proposed, based on tenets of Comprehensive Geriatric Assessment (CGA) and called “multidimensional model”.^18^ This model, using the Multidimensional Prognostic Index (MPI) as assessment tool,^19^ has been applied in different settings and populations including community‐dwelling subjects in whom was estimated a prevalence of multidimensional frailty of 13.3%.^20^ Using its largely demonstrated clinimetric capacities,^18^ the MPI has proven excellent accuracy in predicting several negative outcomes (e.g. mortality, hospitalization and falls) also in community‐based setting.^21^, ^22^, ^23^ Therefore, in this study, we aimed to explore clinical course of frailty condition, as measured by the MPI tools, over 1 year during COVID‐19 pandemic in a cohort of community‐dwelling older adults to understand how COVID‐19 directly and indirectly impacted on frailty condition.

## 2 METHODS

### 2.1 Study population

The PRESTIGE project (Involved and Resilient: Aging in Genoa) is a prospective, observational study aimed to explore frailty and social vulnerability in community‐dwelling older residents in the metropolitan area of Genoa, Italy. We included subjects: (1) aged 65 years or over; (2) community‐dwellers who attended the University of the Third Age (U3A – an international movement whose aim is encouraging the education of retired members of the community) in Genoa according to a lifelong learning program for subjects in their “third age” of life; (3) without acute clinical conditions and (4) able to provide informed consent. Participants were enrolled between November 2019 and February 2020 following the World Medical Association's 2008 Declaration of Helsinki and the guidelines for Good Clinical Practice. Reporting of the study conforms to broad EQUATOR guidelines.^24^

**FIGURE 1:**
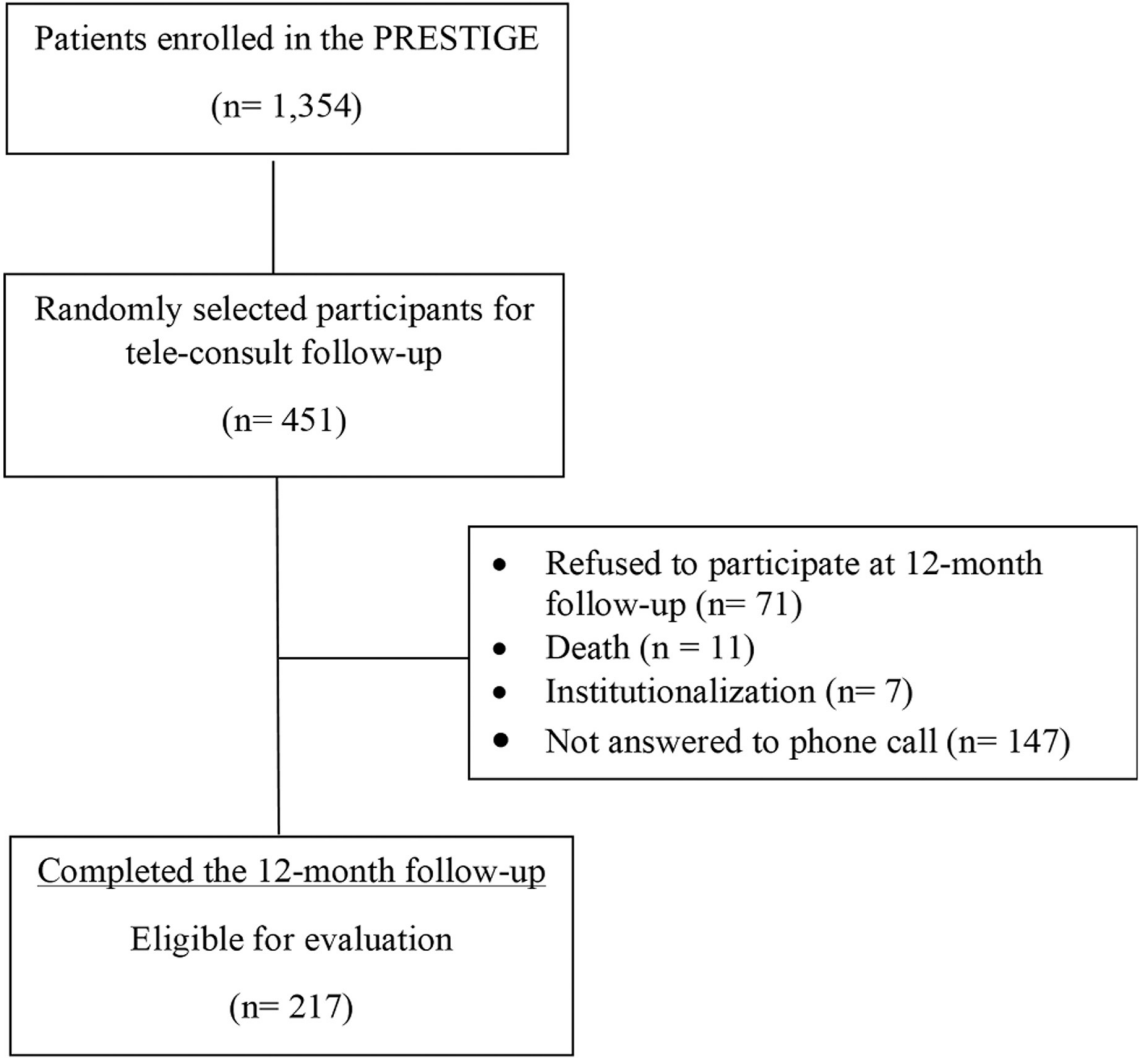
Flow of study participants through the study

From the original sample of 1354 subjects, 451 were randomly selected to undergo tele‐consult follow‐up at 12 months. Out of 380 subjects who agreed to participate, 217 completed the follow‐up and were eligible for this post hoc analysis (Figure 1). The included participants were interviewed by phone call at 12‐month follow‐up between November 2020 and February 2021, in order to reassess frailty condition and to gather information related to eventual Sars‐CoV‐2 infection. Considering that COVID‐19 emerged in Italy in March 2020, we were able to estimate the incidence of COVID‐19 positivity during the first and second pandemic waves in Liguria region. At follow‐up were also collected information regarding hospitalizations.

The Ethical Committee of Department of Education of the University of Genoa (DISFOR), Genoa, Italy, approved the present study. All participants read and signed the informed consent form and all participants' records, and personal information were rendered anonymous before statistical analysis.

### 2.2 Frailty

Frailty assessment was based on multidimensional model of frailty using Multidimensional Prognostic Index (MPI) tools.^18^ In this study we adopted Self‐Administered MPI Short Form (SELFY‐MPI‐SF) ^25^ and Telephone‐administered MPI (TELE‐MPI) ^26^ both deriving from the standard Multidimensional Prognostic Index (MPI).^19^ These have been developed and validated to extend the spectrum of application of multidimensional approach to frailty, based on the principles of Comprehensive Geriatric Assessment (CGA), in more specific settings as community/general practice (SELFY‐MPI‐SF) ^25^ and telehealth (TELE‐MPI).^26^ Both tools showed strong agreement with standard MPI.^25^, ^26^

#### 2.2.1 Self‐administered MPI short form (SELFY‐MPI‐SF)

The SELFY‐MPI‐SF was used to evaluate frailty at baseline, by combining information on the following eight domains, assessed through eight self‐administered scales;^25^

- *Functional status*: evaluated by the Barthel Activities of Daily Living (ADL) sub‐scale which explores the level of dependence/independence in six daily personal care activities such as feeding, bathing, personal hygiene, dressing, faecal and urinary continence and toilet use;^27^, ^28^
- *Mobility*: evaluated by the Barthel mobility sub‐scale assessing the abilities to getting in and out of bed/chair, walking, and going up and down the stairs;^27^, ^28^
- *Independence in the instrumental activities of daily living* (IADL): assessed through the self‐administered version of Lawton's IADL scale exploring the independence in eight activities such as telephone use, grocery shopping, meal preparation, housekeeping, laundry, travel, medication, handling finances;^29^
- *Cognitive status*: measured with the Test Your Memory (TYM), a 10‐task self‐administered test that explores following cognitive domains: orientation, ability to copy a sentence, semantic knowledge, calculation, verbal fluency, similarities, naming, visuo‐spatial abilities and recall of a previously copied sentence.^30^ Final score ranges between 0 and 50, with lower scores indicating worst cognitive function;^30^
- *Nutritional status:* measured with the self‐administered version of the Mini Nutritional Assessment Short‐Form (MNA‐SF), which collects information on anthropometric measures (body mass index and weight loss), decline in food intake, mobility, recent psychological stress and neuropsychological problems;^31^
- *Number of drugs* regularly taken by the subject;
- *Comorbidity*: evaluated by number of pathologies requiring chronic drug therapy, among the first 13 categories of the Cumulative Illness Rating Scale (CIRS);^32^
- *Co‐habitation status* including living alone, in an institution or with family members.

For each domain, a tripartite hierarchy is adopted based on conventional cut‐off points: a score of 0 indicates no problems, 0.5 minor problems and 1.0 major problems. The average of all these eight domains corresponds to SELFY‐MPI‐SF score, with values ranging between 0 and 1 (the higher the score, the greater the degree of frailty).^25^ Also, according to the previously established MPI categories,^19^ the SELFY‐MPI‐SF was expressed as three grades of risk: SELFY‐MPI‐SF grade 1 low risk (values ≤0.33), SELFY‐MPI‐SF grade 2 moderate risk (values between 0.34 and 0.66) and SELFY‐MPI‐SF grade 3 high risk (MPI value >0.66).^25^ We defined as pre‐frail/frail subjects those with SELFY‐MPI‐SF grades 2 or 3, conversely those with SELFY‐MPI‐SF grade 1 were identified as robust participants.

#### 2.2.2 Telephone‐administered MPI (TELE‐MPI)

The TELE‐MPI was collected 12 months apart from baseline assessment by contacting participants with phone call, from November 1, 2020 to February 28, 2021. Similarly to the SELFY‐MPI‐SF domains, the TELE‐MPI considered the same eight areas of CGA.^26^ The only domains evaluated in different way compared to the SELFY‐MPI‐SF were mobility and cognition. Mobility was evaluated inquiring about: (1) the abilities to transfer from bed to chair or wheelchair, (2) walking at least ten feet without any assistance and (3) going up and down the stairs without assistance.^21^ If the subject is able to perform the task was assigned 1 point.^21^ Cognitive performances were assessed using the Short Portable Mental Status Questionnaire (SPMSQ) scale with a score ranging from 10 (worst score) to 0 (best score).^33^ Despite these differences in these two scales, the same tripartite hierarchy was adopted to assign a score of: 0 (no problems), 0.5 (minor problems) and 1.0 (major problems), using previously proposed scale‐specific cut‐off values.^26^ Thus, the sum of the scores assigned to each domain was divided by 8 to obtain a final TELE‐MPI risk score ranging ranging between 0 and 1 (the higher the score, the greater the degree of frailty).

### 2.3 Statistical analysis

Descriptive statistics were expressed for continuous variables as mean and standard deviation (SD) or median and interquartile range (IQR) and for discrete variables as absolute and relative frequencies (percentages) by MPI category (MPI grade 1 vs MPI grades 2–3). Independent sample t‐test or Mann–Whitney U test was used for comparison of continuous variables between groups. Chi‐square and Fisher's exact tests were used to compare categorical factors. Differences in hospitalizations and COVID‐19 cases were analysed using the Cochran–Mantel–Haenszel test. Paired sample t‐tests were used to compare MPI scores at baseline and at 12 months follow‐up among subjects reporting MPI grade 1 or grades 2–3 at baseline with or without Sars‐CoV‐2 infection. Multivariable logistic regression models were developed to identify whether frailty status and Sars‐CoV‐2 infection could predict worsening of frailty condition expressed as difference of MPI scores between 12 months follow‐up and baseline ≥0.1. We selected this cut‐off to identify significant worsening of frailty, based on previous literature showing that increase of 0.1 points of the MPI score was a clinically relevant change associated with increased risk of negative outcomes.^8^, ^22^, ^26^ A two‐tailed significance level at *p* = 0.05 was set for each test. All the analyses were performed using SPSS v26.0 software for Windows (SPSS, Chicago, IL, USA).

## 3 RESULTS

Overall, 217 community‐dwelling older adults (mean age 79.44 ± 7.75 years, range 62–107 years old; females: 49.8%) completed the MPI both at baseline and at 12 months follow‐up. Mean MPI score at baseline was 0.30 (SD: 0.18) with a prevalence of pre‐frail/frail subjects (MPI grades 2–3) of 48.4%. As shown in Table 1, pre‐frail/frail subjects had higher level of functional and cognitive impairment, malnutrition, social isolation and more comorbidities and number of medications compared to those with MPI grade 1 (robust subjects). Flu and anti‐pneumococcal vaccines were not performed in 21.7% and 59% of participants, respectively, with significant lower coverage for pneumococcal vaccination among more pre‐frail/frail subjects (32.4% vs. 49.1% in robusts, *p* = 0.012). Incidence of Sars‐CoV‐2 infection was 12.9%, but was almost five‐time higher among frailer compared to robust subjects (21.0% vs. 5.4%, OR: 4.68, 95% CI: 1.82–12.07, *p* = 0.001). Pre‐frail/frail subjects were also more prone to undergo hospitalizations during the follow‐up compared to robust individuals (26.3% vs. 4.5%, OR: 7.55, 95% CI: 2.70–21.05, *p* < 0.001).

**TABLE 1:** Characteristics of patients by MPI category at baseline

	Overall (n = 217)	MPI grade 1 (n = 112)	MPI grade 2–3 (n = 105)	*p*‐value
Age (years), *mean (SD)*	79.44 (7.75)	76.37 (7.12)	82.71 (7.06)	<0.001
Female, *n (%)*	108 (49.8)	42 (37.5)	66 (62.9)	<0.001
BMI (kg/m^2^), *mean (SD)*	25.53 (4.28)	24.88 (3.62)	26.23 (4.80)	0.020
Diseases, *n (%)*				
Hypertension	119 (54.8)	44 (39.3)	75 (71.4)	<0.001
Cardiac	73 (33.6)	56 (53.3)	17 (15.2)	<0.001
Vascular	52 (24.0)	13 (11.6)	39 (37.1)	<0.001
Respiratory	23 (10.6)	4 (3.6)	19 (18.1)	0.001
Endocrine‐metabolic	48 (22.1)	12 (10.7)	36 (34.3)	<0.001
Upper gastrointestinal	85 (39.2)	29 (25.9)	56 (53.3)	<0.001
Lower gastrointestinal	28 (12.9)	6 (5.4)	22 (21.0)	0.001
Liver	13 (6.0)	3 (2.7)	10 (9.5)	0.034
Kidney	20 (9.2)	4 (3.6)	16 (15.2)	0.003
Genitourinary	30 (13.8)	9 (8.0)	21 (20.0)	0.011
Musculoskeletal	54 (24.9)	12 (10.7)	42 (40.0)	<0.001
Ophtalmological and otorhinolaryngology	40 (18.4)	6 (5.4)	34 (32.4)	<0.001
Neurological	25 (11.5)	2 (1.8)	23 (21.9)	<0.001
Psychiatric	25 (11.5)	7 (6.3)	18 (17.1)	0.012
No flu vaccination 2019, *n (%)*	47 (21.7)	30 (26.8)	17 (16.2)	0.058
No anti‐pneumococcal vaccination, *n (%)*	128 (59.0)	57 (50.9)	71 (67.6)	0.012
MPI, *mean (SD)*	0.30 (0.18)	0.15 (0.09)	0.46 (0.09)	<0.001
ADL, *median (IQR)*	0 (1)	0 (0)	2 (8)	<0.001
Barthel mobility, *median (IQR)*	0 (0)	0 (0)	0 (13)	<0.001
IADL, *mean (SD)*	6.57 (2.01)	7.63 (0.74)	5.44 (2.31)	<0.001
CIRS, *n (%)*	2 (3)	1 (1)	4 (3)	<0.001
TYM, *mean (SD)*	41.53 (7.81)	45.00 (3.94)	37.83 (9.13)	<0.001
MNA‐SF, *mean (SD)*	11.58 (2.44)	12.71 (1.77)	10.37 (2.49)	<0.001
Number of medications, *median (IQR)*	4 (5)	2 (3)	7 (5)	<0.001
Living alone, *n (%)*	108 (49.8)	36 (32.1)	72 (68.5)	<0.001
Hospitalizations, *n (%)*	26 (13.6)	5 (4.5)	21 (26.3)	<0.001
COVID‐19 positivity, *n (%)*	28 (12.9)	6 (5.4)	22 (21.0)	0.001

Abbreviations: ADL, activities of daily living; BMI, body mass index; CIRS, Cumulative Illness Rating Scale; IADL, instrumental activities of daily living; MNA‐SF, Mini Nutritional Assessment Short Form; MPI, Multidimensional Prognostic Index; SD, standard deviation; TYM, Test Your Memory.

**FIGURE 2:**
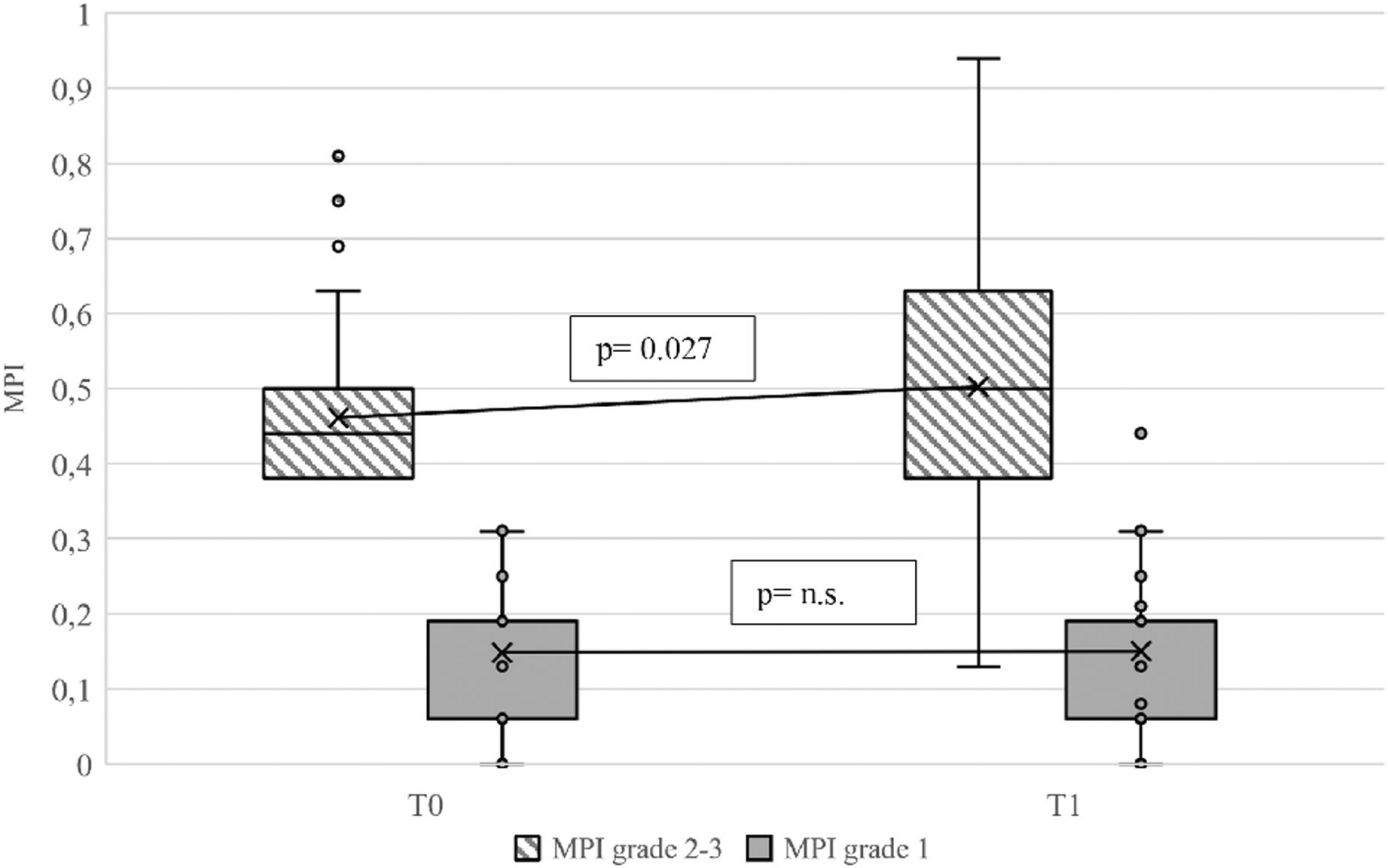
Trajectories of multidimensional frailty based on MPI category at baseline. Center lines show the medians; box limits indicate the 25th and 75th percentiles; whiskers extend 1.5 times the interquartile range from the 25th and 75th percentiles; cross represents sample mean; data points are plotted as open circles. T0 = baseline; T1 = 12 months; MPI = Multidimensional Prognostic Index

While robust subjects at baseline remained on average stable during the follow‐up, indeed no subject evolved toward a pre‐frailty/frailty condition, we found that pre‐frail/frail older adults underwent significant deterioration of MPI score during 12 months (0.46 ± 0.09 at baseline vs 0.50 ± 0.17 at 12 months, *p* = 0.027) (Figure 2). Indeed, in the multivariable analysis, adjusting for age, gender, BMI, multimorbidity (3 or more chronic diseases), flu and anti‐pneumococcal vaccination and COVID‐19 positivity, pre‐frail/frail subjects at baseline experienced a significant higher risk of further worsening of frailty condition (adjusted odd ratio (aOR): 13.60, 95% confidence interval (CI): 4.01 to 46.09, *p* < 0.001) compared to robust subjects (Table 2, Table S1). Pre‐frail/frail subjects even though not‐infected by Sars‐CoV‐2 experienced a significant worsening of multidimensional frailty at 12 months follow‐up (MPI: 0.45 ± 0.08 at baseline vs 0.51 ± 0.17 at 12 months, *p* = 0.005). In the multivariable analysis, adjusting for age, gender, BMI, multimorbidity and flu and anti‐pneumococcal vaccination, pre‐frail/frail older adults both non‐infected (aOR: 14.84, 95% CI: 4.26 to 51.74, *p* < 0.001) and infected by Sars‐CoV‐2 (aOR: 12.77, 95% CI: 2.66 to 61.40, *p* = 0.001) had significantly greater risk of further worsening of frailty condition compared to robust and non‐infected subjects (Table 3, Table S2). Older adults who were robust at baseline were more likely to experience a worsening in ADL domain, whereas among frail/pre‐frail subjects the domains that contributed more to further MPI worsening were loss of IADL, poor mobility, cognitive impairment and malnutrition (Table S3).

**TABLE 2:** Predictors of worsening of frailty condition during COVID‐19 pandemic

Independent variable	Worsening of frailty status (ΔMPI ≥0.1)*		
	Adjusted OR	95% CI	*p*‐value
Age ≥ 75years	3.53	1.10–11.36	0.035
Female	2.01	0.91–4.43	0.085
Multimorbidity (≥3 chronic diseases)	0.30	0.10–0.90	0.032
COVID‐19 positivity	1.01	0.35–2.86	0.989
MPI grade 1	REF		
MPI grades 2–3	13.60	4.01–46.09	<0.001

*Note*: *Model adjusted also for BMI, flu vaccination and anti‐pneumococcal vaccination.

Abbreviations: ΔMPI, difference of MPI scores between 12 months follow‐up and baseline; CI, confidence interval; OR, odd ratio; MPI, Multidimensional Prognostic Index.

**TABLE 3:** Risk of worsening of frailty condition according to frailty status at baseline and COVID‐19 positivity

Independent variable	Worsening of frailty status (ΔMPI ≥0.1)*		
	Adjusted OR	95% CI	*p*‐value
Robust without COVID‐19 positivity	REF		
Pre‐frail/frail without COVID‐19 positivity	14.84	4.26–51.74	<0.001
Robust with COVID‐19 positivity	2.53	0.23–27.99	0.449
Pre‐frail/frail with COVID‐19 positivity	12.77	2.66–61.40	0.001

*Note*. ^*^Model adjusted for age, gender, BMI, multimorbidity (3 or more chronic diseases), flu vaccination and anti‐pneumococcal vaccination.

Abbreviations: ΔMPI, difference of MPI scores between 12 months follow‐up and baseline; CI, confidence interval; OR, odd ratio; MPI, Multidimensional Prognostic Index.

## 4 DISCUSSION

In this cohort of community‐dwelling older adults assessed immediately before and during COVID‐19 pandemic in Italy, we found that pre‐frail/frail subjects independently by age, gender and occurrence of Sars‐CoV‐2 infection and several other potential confounders had significant higher risk to experience further worsening of frailty condition after 1 year.

Solid evidence showed that frailty among hospitalized patients with Sars‐CoV‐2 infection was associated with higher risk of more severe forms of the disease, delirium, and death.^6^, ^34^, ^35^ Moreover, levels of frailty condition pre‐infection have been associated with increased care needs after hospitalization and poorer long‐term survival also regardless of features of acute infection.^12^, ^13^, ^34^ Parallelly, frailty has been deemed as a criterion for less aggressive approaches. Indeed, among older adults resident in long‐term care the COVID‐19 positivity and the presence of frailty condition were associated with a de‐escalation of care plans.^36^ Frailty assessed by Clinical Frailty Scale (CFS) has been shown a better predictor of patient outcome compared to chronological age and comorbidities.^37^ However, it has been questioned the reliability of CFS to adequately capture frailty condition.^38^ Results from studies conducted in different settings, including community, confirmed that frailty, measured through multidimensional approach, was associated with a significant higher risk of negative health outcomes.^18^ Higher MPI among older patients hospitalized in acute wards for COVID‐19 disease, as well among long‐term care and nursing home residents during COVID‐19 outbreak, were strong predictors of mortality risk and each 0.1 increase of the MPI score was associated with almost 40% higher probability of death.^8^, ^39^, ^40^ Our data might suggest that impact of COVID‐19 pandemic on frailty condition, in frail older adults, is largely independent by direct effect of virus. Consistently, growing evidence claim the attention on burden of indirect effects of COVID‐19 (i.e., psychological distress, cognitive impairment, malnutrition and physical inactivity), which translate on multidimensional well‐being.^15^ Then, it is reasonable thinking, even more so during this pandemic, that only a CGA‐based approach is qualified to really capture and track changes of frailty condition.

There is still a paucity of evidence on the effects of COVID‐19 outbreak on frailty condition among community‐dwelling older adults. Here we showed that, based on the pre‐pandemic frailty status, older adults experienced different trajectories of frailty during lockdown measures, independently by occurrence of COVID‐19 infection. Consistently, in a population of community‐dwelling frail older adults with hypertension, it has been observed a consensual impairment of physical and cognitive performances during COVID‐19 pandemic.^41^ In a Japanese cohort of older adults assessed during COVID‐19 outbreak, it has been estimated a transition rate from non‐frailty to frailty over 6 months of roughly 10%.^42^ Another study showed an increase of prevalence of social frailty with 10.7% of subjects who converted from robust to social frailty during one‐year follow‐up before and after the declaration of the state of emergency.^43^ This transition seemed to be associated with exacerbation of depressive symptoms, but not with physical and cognitive functions.^43^ Moreover, in a prospective study conducted between May and October 2020 among older adults in England and Spain, it has been observed a reduction of frailty as the restriction measures become less stringent,^44^ suggesting that such effect of pandemic on frailty status might be potentially reversible.^16^

It has been questioned the role of greater biological and social vulnerability in older adults for higher predisposition to Sars‐CoV‐2 infection,^45^ but few studies explored on a population‐based level the risk associated to being frail during COVID‐19 pandemic.^46^, ^47^ In a report from the UK Biobank conducted on 383,845 subjects, frailty status before COVID‐19 pandemic, assessed by both phenotypic and accumulation of deficits models, was associated with roughly two‐time higher risk of severe COVID‐19 infection resulting in hospital admission and death.^46^ Another study carried out on 241 community‐dwelling older adults from the SarcoPhAge cohort, showed that frailty, assessed by Fried criteria, was associated with seven‐time higher risk of Sars‐CoV‐2 infection.^47^ Here, we found an overall incidence of COVID‐19 positivity of 12.9%, which was roughly doubled in pre‐frail/frail subjects. However, independently by Sars‐CoV‐2 infection, we observed a significant worsening of frailty condition only in those subjects who were pre‐frail/frail before COVID‐19 outbreak.

This study has also some limitations that should be disclosed. First, having only two timepoints to assess frailty condition may have limited our ability to accurately capture the trajectories of frailty. Given the potential reversibility and fluctuation of this condition, we cannot state if the observed worsening of frailty status in pre‐frail/frail subjects was a real continuous trend. Therefore, planning an extension of study follow‐up could be essential to address this issue. Second, the COVID‐19 positivity may be underestimated because was self‐reported by patients. Indeed, particularly during the first pandemic wave, some pauci‐symptomatic cases might have been passed unrecognized given also the difficulties in provision of diagnostic tests. Third, to assess multidimensional frailty we used two different tools (i.e., SELFY‐MPI‐SF and TELE‐MPI), but both have been developed from the standard MPI with which showed strong agreement sharing the same explored domains and the same algorithm for calculation, with a mean difference between the MPI and each of two derived tools lower than two decimal points.^25^, ^26^ Therefore, we believe that our results can be poorly affected by this methodological difference. Fourth, additional confounding factors could have been included in our analyses such as severity of COVID‐19 disease, length of quarantine and presence of social support during COVID‐19 disease. Indeed the worsening of frailty condition could be strictly dependent by immunological status of the subjects and therefore the capacity of virus clearance, the duration of hospitalization and isolation, the availability of a caregiver or social services. Fifth, our results, due to relatively high loss at follow‐up, might suffer from selection bias. However the baseline characteristics (e.g., age, gender, comorbidities, SELFY‐MPI‐SF) of subjects who remained in the longitudinal study were overlapping with those who did not complete the follow‐up. Finally, this study was performed only on a relatively small population from a specific geographic area which has a high density of older adults living in the community. Thus, for example, we were not able to differentiate between prefrail and frail subjects, and the generalizability of these findings might be limited and should be verified in larger multicenter studies. Moreover given the post hoc nature of the analysis, the risk of further worsening of multidimensional frailty in pre‐frail/frail subjects during COVID‐19 pandemic needs to be confirmed in ad hoc‐designed studies.

## 5 CONCLUSIONS

Effects of COVID‐19 pandemic among community‐dwelling pre‐frail/frail individuals are far beyond the mere infection and disease, but also might determine a significant deterioration of frailty status. More efforts must be taken to early recognize and manage frailer subjects, to avoid progression toward irreversible disability. Future studies should better define the frailty trajectories testing whether the slope of increase of multidimensional frailty, or reaching a specifical threshold of MPI score, can determine differences in short‐ and long‐term outcomes for community‐dwelling older adults.

## AUTHOR CONTRIBUTIONS

AP: Conceptualization, funding acquisition, supervision, writing – review & editing; CC: methodology, formal analysis, writing – original draft preparation; SZ: data curation, project administration, writing – review & editing; SP: investigation, writing – review & editing; BS: investigation, writing – review & editing; CP: investigation, writing – review & editing; ET: investigation, writing – review & editing; NV: investigation, writing – review & editing; EZ: investigation, writing – review & editing; CT: investigation, writing – review & editing; CS: investigation, writing – review & editing; AC: conceptualization, investigation, writing – review & editing.

## FUNDING INFORMATION

This work was supported by Fondazione CARIGE (“Stronger, less frail” GRANT 2018).

## CONFLICT OF INTEREST

The authors declare that they have no competing interests.

## ETHICAL APPROVAL AND CONSENT TO PARTICIPATE

The Ethical Committee of Department of Education of the University of Genoa (DISFOR), Genoa, Italy approved the present study on 5 September 2019; study number 030.

## CONSENT FOR PUBLICATION

Not applicable.

## Supporting information


Table S1‐S3

## APPENDIX 1

### The PRESTIGE Study Investigators

Marina Barbagelata, Lisa Annunziata Cammalleri, Romina Custureri, Simone Dini, Marcella Fama, Paola Giannoni, Valeria Pandolfini, Annamaria Piana, Alessandra Pinna, Martina Vigo, Erica Volta
