# Supplementary material for: Frailty trajectories in community‐dwelling older adults during COVID‐19 pandemic: The PRESTIGE study
Source: Eur J Clin Invest. 2022 Jul 28:e13838. Online ahead of print. doi: 10.1111/eci.13838 (PMC9350279; doi:10.1111/eci.13838)
Supplement: Supplementary file 1 — Table S1‐S3 [file ECI-9999-0-s001.docx]

**Supplementary Table 1. Proportion of subjects experiencing during follow-up a worsening in eight MPI domains based on MPI category at baseline**

| **Worsening domain*** | **Overall**  **(n= 217)** | **MPI grade 1**  **(n= 112)** | **MPI grade 2-3**  **(n= 105)** | **p-value** |
| --- | --- | --- | --- | --- |
| ADL, *n (%)* | 149 (68.7) | 112 (100) | 37 (35.2) | <0.001 |
| Mobility, *n (%)* | 40 (18.4) | 4 (3.6) | 36 (34.3) | <0.001 |
| IADL, *n (%)* | 35 (16.2) | 2 (1.8) | 33 (31.4) | <0.001 |
| Comorbidities, *n (%)* | 16 (7.5) | 10 (8.9) | 6 (5.7) | 0.365 |
| Cognition, *n (%)* | 21 (9.9) | 6 (5.4) | 15 (14.3) | 0.026 |
| Nutrition, *n (%)* | 42 (19.6) | 6 (5.4) | 36 (34.3) | <0.001 |
| Polypharmacy, *n (%)* | 15 (6.9) | 7 (6.3) | 8 (7.6) | 0.691 |
| Social status, *n (%)* | 7 (3.2) | 4 (3.6) | 3 (2.9) | 0.766 |

*Note.* *Number of subjects experiencing a transition from lower to higher risk category in the tripartite hierarchy of MPI domains. ADL= activities of daily living; IADL= Instrumental Activities of daily Living; MPI= Multidimensional Prognostic Index.

**Supplementary Table 2. Predictors of worsening of frailty condition during COVID-19 pandemic in a fully adjusted model***

| Independent variable | Worsening of frailty status (ΔMPI ≥0.1)* | | |
| --- | --- | --- | --- |
|  | **Adjusted OR** | **95% CI** | **p-value** |
| Age ≥ 75y | 4.21 | 1.25 – 14.19 | 0.021 |
| Female | 1.79 | 0.80 – 4.01 | 0.153 |
| Multimorbidity (≥3 chronic diseases) | 0.26 | 0.09 – 0.82 | 0.021 |
| COVID-19 positivity | 1.22 | 0.42 – 3.53 | 0.714 |
| MPI grade 1 | REF |  |  |
| MPI grade 2-3 | 19.56 | 4.68 – 81.66 | <0.001 |

*Note.* *Model adjusted also for BMI, flu vaccination and anti-pneumococcal vaccination, MNA-SF at baseline, number of drugs at baseline, and living alone. ΔMPI= difference of MPI scores between 12 months follow-up and baseline; BMI= body mass index; CI= confidence interval; OR= odd ratio; MNA-SF= Mini Nutritional Assessment Short Form; MPI= Multidimensional Prognostic Index.

**Supplementary Table 3. Risk of worsening of frailty condition according to frailty status at baseline and COVID-19 positivity in a fully adjusted model***

| Independent variable | Worsening of frailty status (ΔMPI ≥0.1)* | | |
| --- | --- | --- | --- |
|  | **Adjusted OR** | **95% CI** | **p-value** |
| Robust without COVID-19 positivity | REF |  |  |
| Pre-frail/frail without COVID-19 positivity | 20.99 | 4.90 – 89.89 | <0.001 |
| Robust with COVID-19 positivity | 2.59 | 0.24 – 27.56 | 0.430 |
| Pre-frail/frail with COVID-19 positivity | 22.22 | 3.66 – 134.91 | 0.001 |

*Note.* ^*^Model adjusted for age, gender, BMI, multimorbidity (3 or more chronic diseases), flu vaccination and anti-pneumococcal vaccination. ΔMPI= difference of MPI scores between 12 months follow-up and baseline; BMI= body mass index; CI= confidence interval; OR= odd ratio; MNA-SF= Mini Nutritional Assessment Short Form; MPI= Multidimensional Prognostic Index.
